# The Faraday Scalpel: Electrochemical Nerve Lesioning Mechanisms Studied in Invertebrate Models

**DOI:** 10.1002/advs.202523797

**Published:** 2026-03-17

**Authors:** Petra Ondráčková, Jan Švec, Marie Jakešová, Jiří Ehlich, Imrich Gablech, Eric Daniel Głowacki

**Affiliations:** ^1^ Bioelectronics Materials and Devices Laboratory Central European Institute of Technology Brno University of Technology Brno Czech Republic; ^2^ Institute of Scientific Instruments of the CAS Brno Czech Republic

**Keywords:** bioelectronics, biological electrochemistry, electrosurgery, nerve cuff electrodes, tissue ablation

## Abstract

Electrical lesioning of nervous tissue is a common surgical intervention and traditionally is carried out using high‐amplitude high‐frequency currents. These procedures ablate tissue via an irreversible thermocoagulation or electroporation mechanism. In this work, we explore an alternative concept of achieving lesioning using lower‐amplitude direct currents (DC). DC is necessarily accompanied by faradaic reactions, which can lead to local chemical changes that affect nervous tissue. We elucidate the electrochemical mechanisms behind DC nerve lesioning using two disparate invertebrate models: the leech (*Hirudo verbana*) and the locust (*Locusta migratoria*). These represent convenient low‐cost systems for investigating the effects of DC on nerve functionality with simultaneous in situ electrochemical characterization. Using thin film platinum electrodes, we find that discrete electrochemical processes and associated current magnitudes lead to different outcomes. The lowest current density regime leading to lesioning is cathodic < 100 µA/cm^2^, corresponding to the oxygen reduction reaction (ORR). ORR leads to oxygen depletion near the electrode surface, thus causing hypoxic lesioning. Using positive and negative control experiments, we confirm this novel cathodic hypoxia lesioning mechanism. By using the conducting polymer PEDOT, which favors ORR with hydrogen peroxide as the product, we find that nerve lesioning proceeds with higher efficiency than with platinum, with hydrogen peroxide toxicity as the primary mechanism leading to lesioning. Higher‐level cathodic DC (> 100 µA/cm^2^) corresponds to water electrolysis and leads to more rapid nerve lesioning via local alkalization. Anodic DC also causes rapid nerve lesioning. We find that the current‐induced damage apparently is not related to pH changes or water electrolysis, but likely to chloride oxidation and production of reactive chlorine species. Overall, these results reveal critical current densities that can damage nervous tissue via disparate electrochemical mechanisms. These findings lay a foundation for understanding cathodic and anodic DC current effects on neural tissues, informing experimental and device design for lesioning in mammals, and serving as a reference for neural interface safety margins.

## Graphical abstract

Direct‐current produces nerve lesioning through discrete electrochemical reactions. Using hypoxia‐sensitive locust nerves and hypoxia‐tolerant leech nerves, we map three injury pathways: cathodic oxygen reduction, cathodic alkalization, and anodic chloride oxidation. These findings establish electrochemical lesioning—the “Faraday Scalpel”—as a precise, low‐power modality distinct from conventional thermal or pulsed‐field lesioning.

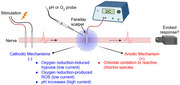

## 1 Introduction

### 1.1 Ablative Electrosurgery

Surgical lesioning of nervous tissue is an intervention for the treatment of neurological disease. The most prominent example is resection of seizure foci in patients with drug‐refractory epilepsy. Localized lesioning of nervous tissue is used in the treatment of some movement disorders and is explored in the context of psychiatric disorders. Aside from simple surgical resection with fine tools, more precise techniques exist, especially for targeting difficult‐to‐reach targets located deep in tissue. Radiofrequency thermocoagulative (aka electrocautery) ablation is a common method, whereby electrical power is dissipated thermally between two electrodes applied to the target region [1, 2, 3]. Photothermal laser techniques, introducing high optical power via implanted fibers, promise higher focality, though the thermal mechanism of action similarly destroys all cell types [4, 5]. Both methods are standard of care in epileptic focus removal. Though often leading to seizure‐free outcomes for patients [6], such procedures indiscriminately destroy tissue, ablating all cell types, including blood vessels [7]. This leads to relatively large lesions with a low degree of postoperative regeneration. Similar electro‐lesioning is also used in the treatment of disorders of the peripheral nervous system, like neuromas, chronic pain, and spastic conditions. Lesioning, which produces shorter‐term nerve failure (on the scale of days to weeks), is often termed “nerve conduction block”, while longer‐term effects are typically labeled as “ablative” surgery. Significant semantic overlap exists between these terms. For clarity, in this paper, we are studying functional failure in nerve function, and thus we will generally term this as “lesioning” or “nerve failure”. Overall, regardless of the timescale of the desired outcome, there is interest in exploring more gentle lesioning techniques that would spare vasculature and encourage postsurgical regeneration of nervous tissue. Recent approaches using thermal or nonthermal irreversible electroporation demonstrate a higher degree of selectivity in ablating neural tissue while preserving vasculature. Postoperative bleeding remains a risk, and application of extremely high voltages presents an obstacle to regulatory acceptance in clinical applications [7]. In all the aforementioned approaches, energy is delivered to the system in a pulsed way, either by alternating current (AC) or light impulses. The power dissipated is on the order of 1–50 W, and in the case of standard AC thermocoagulative ablation, the tissue temperature is reaching around 80°C, corresponding to extensive protein denaturation [2, 8]. Electrocautery applied to peripheral nerves leads to irreversible damage, which is sometimes followed by slow and incomplete regeneration, and thus has therapeutic potential only in limited use cases [9].

### 1.2 Electrochemical Lesioning

The approach evaluated in this work uses electrical current, but it is fundamentally different than the aforementioned technologies: We consider direct current (DC) conditions, where the maximum applied power is on the order of 10–50 µW. The flow of DC across a physiological electrolyte necessarily corresponds to electrochemical (aka Faradaic) reactions, whereby charge is transferred between electrodes and the physiological electrolyte. Our hypothesis is that electrochemical reactions can lead to the breakdown of cellular homeostasis and ultimately cell death. This notion we designate as electrochemical lesioning, or the Faraday Scalpel. The idea that DC can, via the action of electrochemical reactions and/or electric fields, impede or block neural activity is an old one, reported since the 1960s [10, 11]. In particular, DC neural blocking experiments have been carried out on peripheral nerves. These works focused on the concept of “anodal or cathodal nerve block”, where DC induces sustained hyperpolarization or depolarization of neurons, preventing action potential propagation. In several of these studies, it was reported that overapplication of DC damages nerve tissue (semi‐chronic nerve block); this issue was not explored in depth. In 2011, Ravid et al. [12]. revisited this concept, demonstrating that DC‐induced nerve damage can lead to a transient lesioning / nerve‐block phenomenon lasting weeks, which may be used for the treatment of spastic hypertonus. This 2011 study hypothesized that electrochemical reactions, and in particular pH changes, are likely the mechanism behind this lesioning effect, without direct experimental verification of the reaction mechanism. These previous works present a starting off point and inspiration for the present study. The question our work seeks to answer is which particular electrochemical reaction(s) cause the nerve tissue damage. The literature on neural interface safety generally defines the electrolytic “water window” as the safe margin of operation. Our findings show that surprisingly low currents can cause damage via mechanisms that have often been overlooked and are within the “water window”, namely oxygen reduction reactions (ORR) and chloride oxidation reactions. Conceptually, since these are discrete faradaic reactions, we are naming this kind of effect a Faraday Scalpel, which distinguishes this phenomenon from higher‐power electrical scalpels that are in use today.

### 1.3 Experimental Design of Faraday Scalpel Application in Invertebrate Models

**FIGURE 1:**
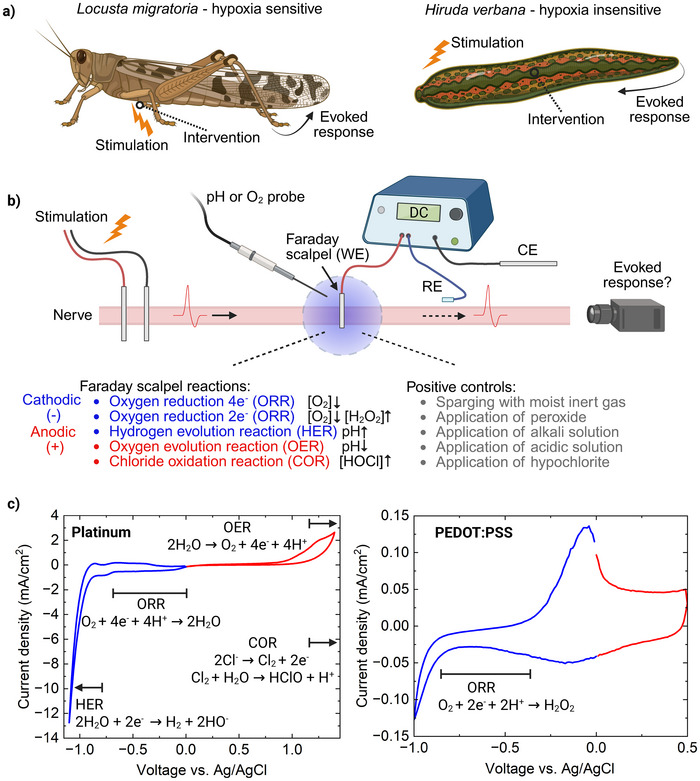
Experimental designs and biological rationale for studying “Faraday Scalpel” DC nerve lesioning mechanisms in two invertebrate models. (a) The two models used in this study differ in their sensitivity to hypoxia. In the locust (*Locusta migratoria*), electrical stimulation of the N5 nerve causes extension of the tibial muscle, which serves as a readout of nerve function. Electrochemical damage to the intervening nerve will give an obvious lack of evoked motor activity. The locust nervous system is sensitive to hypoxia, and thus can serve as a positive test subject for the possible effect of electrochemical oxygen reduction. In the leech (*Hirudo verbana*), the ventral nerve cord is stimulated rostrally to evoke a caudal muscle contraction, which is quantified using a force sensor. The test section of the nerve cord, located between the stimulation and response sites, is accessible for localized electrochemical intervention. The leech nervous system is not sensitive to hypoxia and thus serves as a control subject for the effect of electrochemical oxygen reduction. (b) The general schematic of testing the Faraday scalpel effect. Each model involves stimulation / electrochemical intervention with the Faraday Scalpel/recording of evoked motor movement or failure thereof. Flexible thin‐film DC intervention working electrodes (Faraday Scalpel electrodes) were designed and microfabricated according to the anatomical constraints of each model. Voltage and current are controlled using a potentiostat. Local changes in [O_2_] or pH could be measured with the respective probes. The faradaic reactions we considered, as well as their corresponding positive controls, are listed below the schematic. (c) Cyclic voltammograms with annotated reactions on Pt and PEDOT electrodes in insect physiological medium (10 mV/s). The blue traces at negative voltage values versus Ag/AgCl represent cathodic reactions, while the red traces at positive voltages correspond to anodic reactions. Platinum enables both cathodic and anodic water‐splitting reactions, and catalyzes ORR preferentially via the 4‐electron pathway to water; the 2‐electron pathway is minor. Chloride oxidation is also present on platinum, and competes with water oxidation under anodic polarization. Chloride oxidation is a probable reaction pathway due to high chloride concentration in physiological media. PEDOT:PSS is only relevant for cathodic reactions, where it is highly selective for the 2‐electron ORR to hydrogen peroxide. Any trace HER originates from the substrate metal below the PEDOT:PSS layer. These reaction assignments are based on two recent studies mapping the respective reactions in biological media [19, 20]. Panels a and b were, created in BioRender. Glowacki, E. (2026) https://BioRender.com/si9yuf6.

In this work, we concentrate on identifying the discrete electrochemical reactions that occur under the application of DC in a physiological electrolyte. While the ultimate practical application target is mammalian nervous tissue, we wanted to find a way to systematically probe electrochemical effects on nervous tissue with a low financial and ethical burden, and with the ability to easily conduct in situ electrochemical measurements. The biological models we selected are the locust *Locusta migratoria* and the medicinal leech, *Hirudo verbana* (Figure 1a). These invertebrate models present unique advantages for this study: Besides being robust and low‐cost models, these invertebrates have a well‐understood and relatively simple nervous system [13]. In each case, there is ample anatomical space to introduce electrodes for performing DC lesioning experiments, while simultaneously applying probes to measure local pH and oxygenation. There is a critical biological difference between the two invertebrate models, which is why they were chosen for this study: their nervous system response to hypoxia. In locusts, acute hypoxia or anoxia triggers a rapid shutdown of neural activity via a spreading depolarization and collapse of ionic gradients, causing the insect's nervous system to enter a (semi‐)reversible coma [14]. However, the locust nervous system is not able to survive sustained hypoxia [14]. By contrast, many annelids, including leeches, demonstrate extreme tolerance to low oxygen, capable of maintaining stable electrophysiological function for hours to days in anoxic conditions due to unique metabolic depression pathways [15, 16]. These contrasting responses–the locust's rapid neural response to hypoxia versus the leech's sustained neural function through metabolic depression–help us discriminate between electrochemical effects involving oxygen reduction versus other electrochemical or electrostatic effects. We hypothesize that cathodic hypoxia would lead to nerve conduction failure and tissue damage in the locust, but not in the leech; however, if other non‐oxygen factors like electric field are at play, then cathodic currents should affect both models similarly. The experimental scheme in both animals is of the stimulation/intervention/response type (Figure 1b). In both cases, we utilize flexible thin‐film electrodes microfabricated on ultrathin substrates, allowing conformal contact with the nerve, geometrically tailored for each model. Periodic electrical nerve stimulation is applied to evoke a reproducible motor response: activation of the *extensor tibialis* muscle in locusts [17], and body shortening in leeches [18]. Meanwhile, the intervening section of the nerve is exposed to the DC treatments by the Faraday Scalpel electrode. We also used Clark electrode microsensors to measure in situ oxygen concentration, or a micropipette pH sensor to measure local pH changes. Figure 1b lists the various faradaic reactions that were considered, as well as their corresponding positive controls. Our hypothesis was that low cathodic currents corresponding to oxygen reduction reactions (ORR) would lead to hypoxia, which stops nerve conduction in the locust, but not in the leech. Further, we hypothesized that increased current densities corresponding to water electrolysis would lead to nerve lesioning in both models, due to large pH changes. As our results support the first hypothesis. The second hypothesis is supported only for cathodic currents—the increases in local pH are clearly implicated as the mechanism of nerve lesioning. The hypothesis is falsified for anodic currents, where decreases in pH are small and insufficient to cause damage, and the competing reaction of chloride oxidation apparently is responsible for the observed nerve lesioning.

## 2 Results and Discussion

### 2.1 Electrochemical Reactions at Platinum and PEDOT Electrodes

Sustained flow of DC through a physiological electrolyte requires charge‐transfer reactions (aka Faradaic reactions) to occur at the respective electrodes making up the electrochemical circuit [21]. In this work, we are interested in inducing faradaic reactions in the physiological electrolyte in order to affect homeostasis of the neural tissue. The primary faradaic reactions that are relevant in biological electrolytes are water splitting (oxidative and reductive), oxygen reduction reactions (ORR), and the chloride oxidation reaction. These three types of reactions are present in all biological media. Other redox‐active molecules can also be present in specific settings as well; this work will focus on the aforementioned three types of “universal” faradaic reactions. In implantable bioelectronic devices, platinum (and its alloys like PtIr) is by far the most commonly used and also most electrochemically well‐understood electrode material. Recently, our group [20] and others [22, 23] have mapped in detail the onset potentials of faradaic reactions on medical‐grade platinum electrodes. Another popular electrode material for neural electrodes is Poly(3,4‐ethylenedioxythiophene):polystyrene sulfonate, or PEDOT:PSS for short. PEDOT:PSS has a markedly different electrochemical behavior compared to Pt, as we will discuss in the following. To provide a point of reference for our study, we measured the cyclic voltammogram (CV) of platinum and PEDOT:PSS in insect medium (Figure 1c). In CVs, current is registered while potential is scanned, with increases and peaks in current corresponding to specific electrochemical redox reactions. Under cathodic polarizations, the reaction that occurs at the lowest potential is ORR. The current density of ORR will depend on the electrocatalytic properties of the given electrode material and the quantity of dissolved oxygen in the vicinity of the electrode. The current densities for ORR in normoxic conditions are in the range of 10–300 µA/cm^2^ on Pt, and are apparent at voltages between 0 and −800 mV versus Ag/AgCl [19]. ORR can proceed via 4‐electron reduction to produce water, or 2‐electron reduction to produce hydrogen peroxide. Both reactions would result in consumption of dissolved oxygen and thus hypoxia, and the latter reaction will additionally generate hydrogen peroxide, which may harm tissues. Pt represents a material with a well‐documented electrocatalytic property for 4‐electron reduction, with very little current contributing to peroxide production via the 2‐electron mechanism. We recently studied the oxygen and hydrogen peroxide gradients above DC polarized Pt [20] or PEDOT:PSS [24] electrodes. The oxygen depletion region extends to several hundred micrometers above the electrode surface, even when the surrounding electrolyte is normoxic and exposed to air. For PEDOT:PSS, the concentration gradient of hydrogen peroxide essentially mirrors that of dissolved O_2_, also extending to hundreds of micrometers above the electrode surface. Both hypoxia and peroxide will be evaluated as potential sources of lesioning. Considering more negative potentials, at potentials more negative than −600 mV versus Ag/AgCl, the hydrogen evolution reaction, HER (aka cathodic water electrolysis), clearly dominates [20]. The fact that hydrogen produced on Pt is evident due to the pronounced anodic currents during the backward scan, this is due to H_2_ being oxidized back to H^+^ at the platinum surface [22]. Anodic reactions on platinum are apparent at potentials more positive than +800 mV; at these potentials, oxidative water splitting begins, and around +1000 mV, this reaction coexists with chloride oxidation. Water oxidation releases O_2_ and also protons, thereby leading to acidification of the local environment. Chloride ions are present in relatively high concentrations in biological conditions (on the order of 0.1 m), and thus they are a kinetically competitive substrate for oxidation, producing reactive chlorine in the form of hypochlorite/hypochlorous acid (OCl /HOCl) as the product. We have recently reported on the interplay of reactive chlorine and oxygen evolution at platinum surfaces [20]. Hypochlorite is a very strong oxidant and potent cytotoxin [25]. Under anodic conditions, one can expect acidic pH and/or reactive chlorine to be possible mechanisms of nerve lesioning. As this work will show, the chlorine pathway is likely the dominant mechanism.

As discussed above, ORR can also proceed via a 2‐electron pathway to hydrogen peroxide, H_2_O_2_. Peroxide is also a known cytotoxic compound. Its toxicity varies greatly depending on the environment and the cell type, as various antioxidants and/or enzymes can efficiently break it down [26, 27]. We wanted to test if 2‐electron ORR, which would produce hypoxia and also potentially toxic peroxide as a byproduct, is a potential nerve lesioning mechanism. To this end, we had to choose a suitable electrode material that selectively supports this 2‐electron pathway: the conducting polymer PEDOT. PEDOT is remarkable for having extremely high overpotentials for all the aforementioned reactions discussed on platinum, however, enabling ORR selectively to H_2_O_2_ [24, 28, 29]. PEDOT:PSS can be regarded as a material that uniquely supports hydrogen peroxide production, but does not effectively carry out any of the other reactions mentioned for Pt. Our work will show that the hydrogen peroxide pathway is also a viable nerve lesioning mechanism.

### 2.2 Treatment of the Locust N5 Nerve With Cathodic Faradaic Currents

**FIGURE 2:**
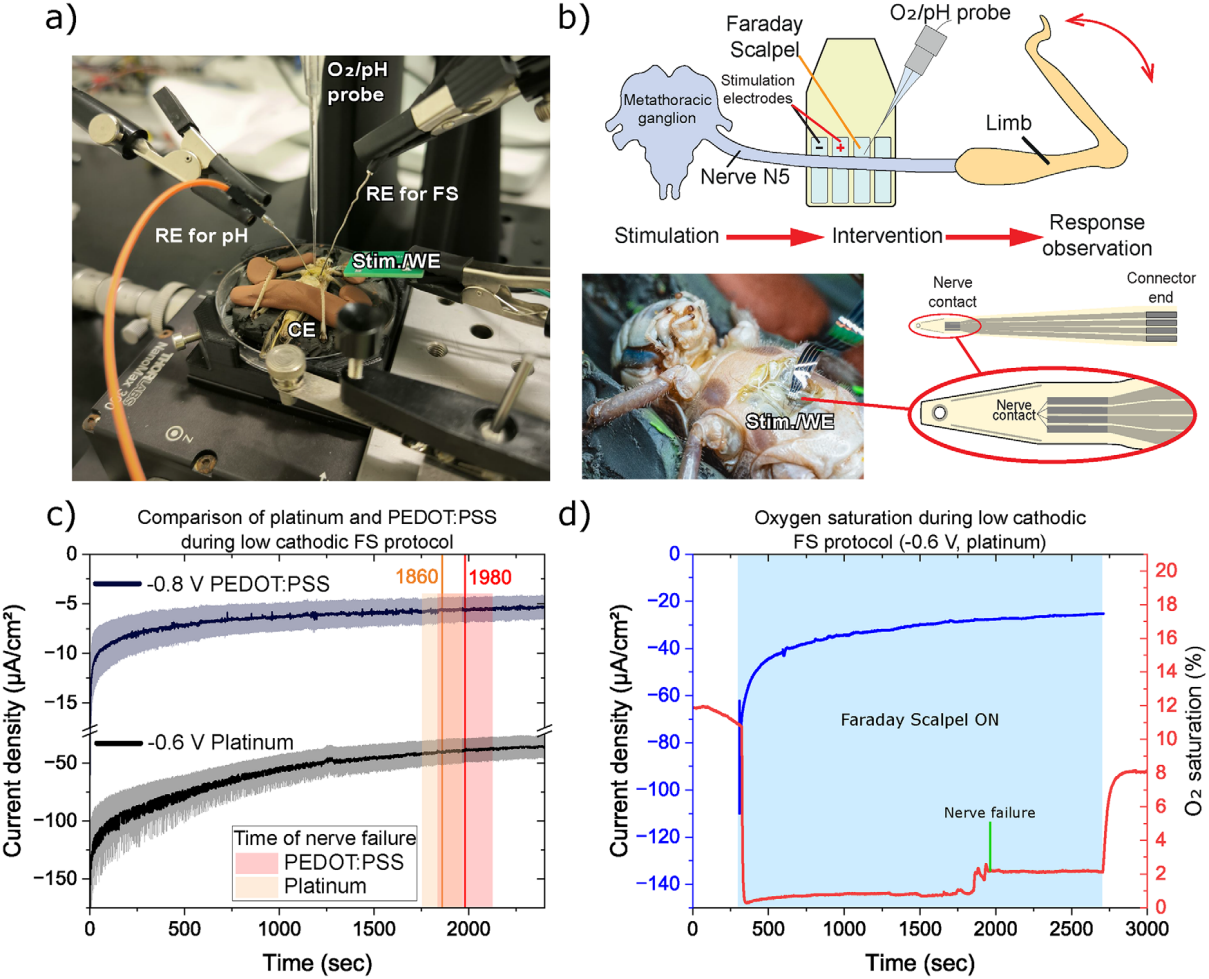
Cathodic oxygen reduction leads to nerve lesioning. (a) Experimental configuration of the locust model for applying the Faraday Scalpel WE to the N5 nerve with in situ measurement of [O_2_] or pH. (b) Stimulation/ intervention/ response experimental setup for measuring nerve conduction failure during DC current application. Flexible probes with either Pt or PEDOT:PSS active electrodes were used. (c) Measurement of nerve failure time after application of cathodic current on Pt or PEDOT:PSS electrodes (for each electrode, *n* = 5 locusts ± S.E.M.). (d) Concurrent oxygen sensor measurement on Pt shows a rapid decrease in O_2_ saturation during cathodic current application, evidencing that nerve lesioning (time point of nerve failure marked with green line) occurs due to hypoxia.

To investigate the effect of electrochemically induced hypoxia on a hypoxia‐sensitive nervous system, we targeted the N5 nerve of *Locusta migratoria*, which controls contraction of the powerful *extensor tibiae* muscle responsible for jumping [17, 30]. Electrical stimulation was used to evoke motor responses (0.016 Hz), and loss of evoked movement served as a functional marker of nerve conduction failure. The setup for this experiment is shown in Figure 2a. When sustained cathodic DC was applied using flexible platinum or PEDOT:PSS thin‐film electrodes (schematic Figure 2b) under potentiostatic control, we observed a time‐dependent sudden loss of nerve conduction. In the low cathodic regime, corresponding to the oxygen reduction reaction (ORR) (For Pt: −0.6 V versus Ag/AgCl; for PEDOT:PSS: −0.8 V versus Ag/AgCl), the *extensor tibiae* muscle ceased to respond after ≈ 30 min of DC application (Figure 2c). The mechanism of nerve failure is apparently different in the case of both electrode materials, however. Concurrent oxygen sensor measurements at the site of cathodic current application confirmed that ORR on Pt leads to a rapid drop in [O_2_] from a baseline value of ≈ 12% to below 3%. (Figure 2d). Due to the size of the hypoxic gradient (100–200 µm above the platinum should is fully depleted) [20, 31], the sensor should reflect the actual oxygen concentration in the nerve tissue. Positive control experiments using local humid nitrogen gas flow confirmed the locust's susceptibility to hypoxia: muscle responses disappeared within (21 ± 4 S.E.M.) minutes of applying nitrogen deoxygenation (Figure S1). We also checked if local pH changes caused by ORR could be a mechanism of tissue damage. Using a local pH microprobe, we observed no measurable pH changes (Figure S2), which is in line with expectations for buffering capacity at these low current densities [20] (in contrast to higher polarizations, as will be discussed in the following section). These findings support the hypothesis that cathodic DC application induces local hypoxia via ORR on Pt, and hypoxia is the mechanism leading to conduction failure. Another observation is the roughly tenfold difference in current between Pt and PEDOT electrodes, yet similar nerve failure times. This would suggest that, in terms of current efficiency (faradaic efficiency), PEDOT:PSS is much more effective. To explain this difference, one has to consider the faradaic reaction differences between the two electrode materials. First, a factor of ×2 in current comes from the fact that Pt catalyzes 4‐electron ORR, while PEDOT:PSS enables 2‐electron ORR. Thus, in terms of deoxygenation faradaic efficiency, PEDOT:PSS will be ×2 better. The rest of the current discrepancy comes from the substantial current contribution from HER. At −600 mV versus Ag/AgCl, HER occurs concurrently with ORR on platinum [20]. However, another important factor is that 2‐electron ORR on PEDOT:PSS will form hydrogen peroxide, H_2_O_2_, as a byproduct. This itself may be cytotoxic [26]. We therefore performed a positive control experiment of H_2_O_2_ application. We found that the addition of 70 µm peroxide solution caused nerve failure. This concentration is well within the range of electrochemically plausible peroxide generation for PEDOT:PSS [19, 24, 28, 32]. This suggests that in systems catalyzing 2e^−^ oxygen reduction (PEDOT electrodes), peroxide toxicity may contribute alongside hypoxia. We next tried to tease apart the relative contributions of hypoxia versus peroxide. First, we observed that during cathodic current application on PEDOT:PSS, the [O_2_] did not drop as in the case of Pt electrodes. This, however, is not unexpected since hydrogen peroxide enzymatically degrades in locust lymph, producing oxygen as a byproduct. However, this would suggest that since oxygen is being regenerated, in fact, peroxide toxicity, and not hypoxia, is the mechanism of nerve lesioning on PEDOT:PSS. To confirm or exclude the peroxide effect, we tested the application of cathodic current on PEDOT:PSS while adding catalase (selectively decomposes peroxide to give O_2_ as a byproduct), or adding pyruvate (decomposes peroxide with CO_2_, acetate, and H_2_O as byproducts). The addition of catalase or pyruvate eliminates the nerve lesioning effect using PEDOT:PSS electrodes. Taken together, these measurements strongly suggest that cathodic peroxide production causes nerve lesioning and hypoxia plays little or no role. Therefore, cathodic nerve lesioning via ORR can take two potential routes, depending on the electrocatalytic properties of the chosen electrode material: Hypoxia from ORR (Pt) or hydrogen peroxide from ORR (PEDOT:PSS).

**FIGURE 3:**
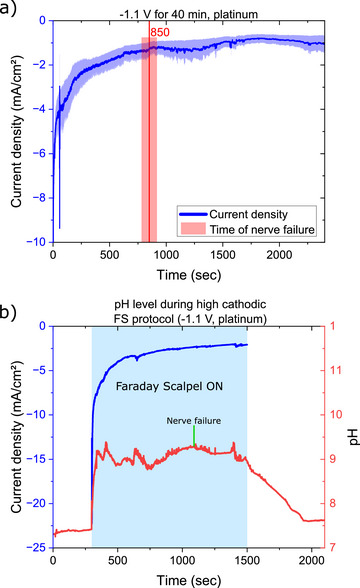
Application of high cathodic currents to the locust N5 nerve causes nerve lesioning, which is attributable to local alkalization. (a) The N5 nerve ceases to respond to stimulation rapidly after application of high cathodic currents. (*n* = 5 locusts ± S.E.M.) (b) Single representative experimental run with simultaneous pH recordings at the working electrode site using a pH microprobe, showing a rapid increase after applying high cathodic current, signaling that alkalization is the reason for nerve conduction failure.

In contrast, at higher cathodic currents on Pt electrodes (≥ −1.1 V versus Ag/AgCl | −1000 µA/cm^2^), which induce water electrolysis and thus will result in local alkalization [20, 22], nerve conduction failure occurred more rapidly, within ≈14 min (Figure 3a). Alkalization is confirmed using a local pH microsensor (Figure 3b). Experiments in which alkaline buffer solutions of increasing pH were applied showed that the N5 nerve ceased to respond to stimulation only after application of a pH 9 solution, supporting the conclusion that alkalization can contribute to N5 nerve failure under these conditions. This observation of alkalization‐dependent nerve damage is consistent with the “water window” picture that has been suggested in the literature before [12]; however, to the best of our knowledge, this is the first direct in situ measurement of this kind.

Importantly, nerve failure in all cases was irreversible, with no recovery of function after 48–72 h of post‐treatment observation. Time‐control experiments in which electrodes were implanted but no current applied showed no nerve damage, confirming the specificity of the electrochemical insult and that damage is not due to mechanical manipulation. These time‐based controls also involved application of the same periodic electrical stimulation that is used in the lesioning experiments. These findings overall demonstrate that relatively modest cathodic currents can lesion peripheral nerves in insects via a non‐thermal, electrochemical mechanism of ORR lesioning, which can act via hypoxia and/or hydrogen peroxide delivery.

### 2.3 Treatment of the Leech Nerve Cord With Cathodic Faradaic Currents

**FIGURE 4:**
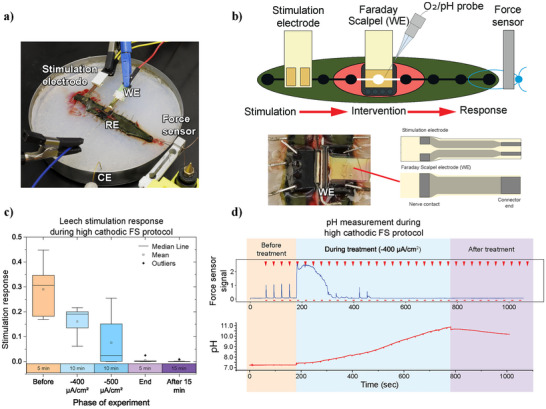
Application of high cathodic currents to the leech nerve cord causes alkalization, which leads to nerve cord conduction failure. (a) Photograph of the leech setup for the Faraday Scalpel. (b) Schematic of the leech experimental setup. The Faraday scalpel electrode wraps around an isolated segment of the nerve cord, and the bipolar stimulation electrode is separate and applied to the rostral section. A force sensor is used to measure evoked movement (full‐body shortening). (c) Quantified evoked force response over the course of experimental time blocks before stimulation, during subsequent cathodic 400 and 500 µA/cm^2^ current density applications, and during time blocks following the current being turned off. n = 5 leeches ± S.E.M. The experimental sequence evidences cathodic induction of nerve failure. (d) In situ pH measurement during a cathodic current application experiment, showing the correlation of loss of evoked response to pH increase. Red triangles mark stimulation time points.

The nerve cord of Hirudo verbana presents an ideal invertebrate model for systematic in situ electrochemical investigation due to its accessibility, size, and robust tolerance to environmental stress [18]. Using a stimulation‐response model similar to that used in the locust, electrical stimulation of the rostral segment evoked consistent caudal muscle contractions, measured via a tension sensor (Figure 4a,b). Cathodic DC was applied to a mid‐cord segment using a microfabricated flexible platinum electrode wrapped around the nerve cord, which was placed inside a custom chamber to facilitate reproducible and well‐defined electrode contact around the whole nerve circumference. Unlike in the locust, low‐level cathodic currents (e.g., −0.8 V, corresponding to ORR) did not lead to any observable degradation of neural conduction even after 1 h of application. These findings are consistent with the known hypoxia tolerance of leech nervous tissue. Positive control experiments using nitrogen or argon gas to deoxygenate the nerve region (Figure S1) failed to produce nerve cord failure, corroborating resistance to hypoxic stress, which is well‐attested in the literature [16, 33, 34]. We also tested the application of hydrogen peroxide at concentrations ranging from 10 nm to 1 mm, but this had no effect on cord failure until 1 mm was applied. A concentration of 1 mm is not practically reachable via electrochemistry with the Faraday Scalpel, however. Therefore, we concluded that neither hypoxia nor hydrogen peroxide would contribute to the leech nerve cord failure. Taking these negative results together, chemical and electrochemical, suggests that with this cathodic current range, nerve lesioning by electric‐field effects alone is not occurring.

Since the lower cathodic currents do not cause any measurable lesioning in the leech, in line with expectations concerning the leech nervous system robustness with respect to hypoxia and reactive oxygen species stress, we proceeded to apply high cathodic treatment corresponding to HER (−1.1 V on Pt). When high cathodic currents, −400 or −500 µA/cm^2^ (corresponding to driving voltages of −1.1 to −1.3 V versus Ag/AgCl) were applied, a clear reduction in evoked muscle force was observed after approximately 10 min (Figure 4c). Using a pH probe at the active site (for these experiments the FS electrode was not completely wrapped around the nerve, to allow access of the pH probe from the top), concurrent pH measurements revealed rapid alkalization of the nerve environment, with local pH rising above 10 during stimulation, consistent with the electrolysis of water and hydroxide ion accumulation (Figure 4d). Control experiments showed that exposure to alkaline buffer solutions of pH ≥10 was sufficient to cause nerve conduction failure in the leech, confirming that alkalization is the primary mechanism of nerve lesioning at high cathodic potentials (Figure S3). Further stimulation cycles were then conducted after the DC current was switched off, and we found no recovery of nerve conduction, at least within a time frame of 60 min. The same lack of reversibility was found with the alkali solution application.

### 2.4 Treatments With Anodic Faradaic Currents

The primary oxidative reaction expected under anodic current application is anodic water splitting, with the evolution of O_2_, accompanied by H^+^ release and thus acidification (Oxygen evolution reaction, OER). Physiological solutions also contain chloride at relatively high concentrations ([Cl^−^] = 135 mm), thus making chloride oxidation a possibility as well, at voltages more positive than 1.1 V versus Ag/AgCl [20]. Additionally, biological solutions contain various organic molecules that can be easily oxidized, producing a sustained anodic current [20]. We did not explore any possibilities of these oxidations or other reactions as contributing to tissue damage.

**FIGURE 5:**
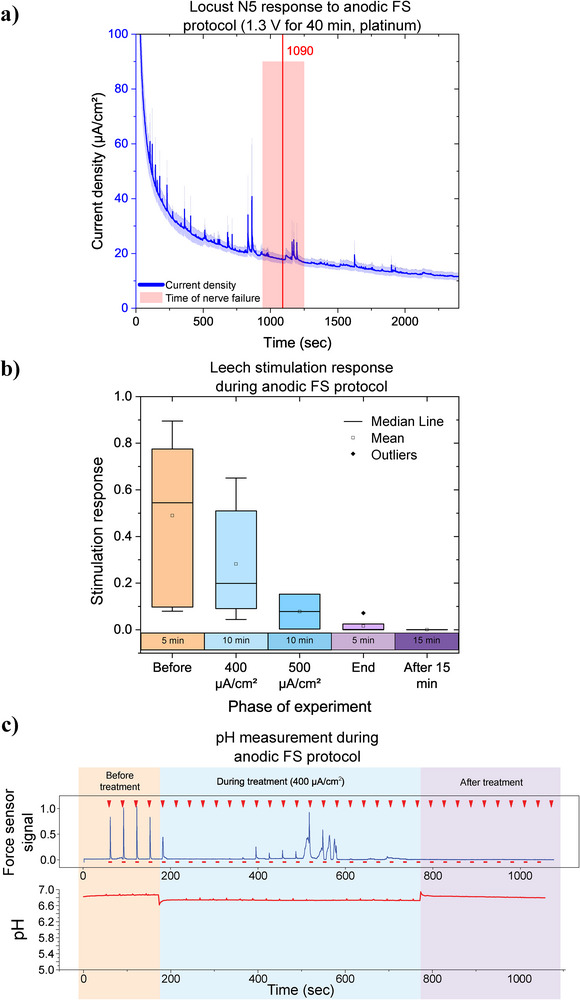
Application of anodic currents in locusts and leeches causes nerve lesioning via a pH‐independent mechanism. (a) Application of +1.3 V versus Ag/AgCl in the locust causes efficient nerve lesioning, n = 5 locusts. ± S.E.M. No pH changes were measurable. (b) Anodic lesioning in leeches requires higher current densities and voltages between +1.3 and 1.6 V versus Ag/AgCl. (c) Superimposed graphs of evoked response (stimulation time points marked with red triangles) measured using the force sensor (upper panel) and in situ pH (lower panel). Local pH changes during anodic current application in leeches are less than one unit, indicating that acidification is not likely the primary mechanism of nerve lesioning.

All anodic experiments were conducted using platinum, due to its relative stability against corrosion upon anodic potential application. In the locust, we found that the N5 nerve was relatively easy to lesion using anodic currents. At +1.3 V versus Ag/AgCl, corresponding to a sustained current of several tens of µA/cm^2^, nerve lesioning happens within ≈15 min (Figure 5a). The leech nerve cord proved to be more robust, and we observed anodic nerve cord failure at 400 or 500 µA/cm^2^ corresponding to 1.3–1.6 V versus Ag/AgCl (Figure 5b). The question is which anodic reaction is responsible for the observed lesioning. We first considered OER, which would lead to local acidification. In the locust, we could not register any measurable pH change during anodic current application (Figure S4). Even in the leech, where the critical lesioning current density was significantly higher than in the locust, the measurable acidification was minor, less than one pH unit (Figure 5c). We conducted a control experiment on the leech of adding progressively more acidic medium, decreasing pH from 7 to 2 (Figure S5). Only values below pH 4 caused a decrease in the evoked response, and such low values were impossible to register during electrochemical treatment. Thus, anodically‐induced pH change is very unlikely to be the cause of nerve lesioning in either model. This rather implicates the chloride oxidation reaction as the source of lesioning. Chloride oxidation produces Cl_2_, which in aqueous conditions reacts to form an equilibrium of hypochlorite/hypochlorous acid (commonly known as chlorine bleach). As a positive control experiment, we treated the locust N5 and the leech nerve cord with various concentrations of hypochlorite, NaOCl (Figure S6). We confirmed that hypochlorite at a concentration of 7 mm (bolus addition) induced muscle contraction activity, an evoked response we also observed during anodic DC application, and that a higher concentration (14 mm) resulted in complete and rapid functional failure of the nerve in both models within 5 min post‐application. Such concentrations, based on measured faradaic efficiency values for the production of hypochlorite on platinum, are not reachable electrochemically. We attempted to quantify the hypochlorite evolution in situ but were unable to do so, as hypochlorite reacts extremely rapidly with organic substrates and decomposes, making the definition of precise in situ concentrations impossible [20]. Determining critical concentration values for hypochlorite toxicity is challenging, since the active chlorine degrades as soon as it enters the biological medium. Determining the critical concentration at the nerve cells is a complicated experimental puzzle. Nevertheless, by virtue of elimination, it would seem that chlorine bleach is a likely mechanism for tissue damage, because acidification can be excluded.

## 3 Discussion and Conclusions

This study demonstrates that DC stimulation can induce robust, irreversible lesioning of nervous tissue through a set of discrete electrochemical reactions, some of which occur well within the classical “water window” typically considered in bioelectronics safety frameworks. By systematically isolating the contributions of oxygen reduction, water electrolysis, and chloride oxidation in two physiologically distinct invertebrate models, we provide a mechanistic map of how faradaic processes disrupt neural function. These findings broaden the understanding of DC‐induced tissue injury and introduce the concept of electrochemical lesioning—the Faraday calpel—as a chemical, non‐thermal alternative to conventional ablative tools. The specific Faradaic reactions, as well as their corresponding positive control experiments, are summarized in Table 1.

**TABLE 1:** Summary of tested DC current regimes along with control experiments and their effects on the neural tissue. The column on the right indicates whether the Faradaic Scalpel (FS) principle can be applied electrochemically or not.

Current regime	Biological model	Nerve lesioning (failure of nerve function)	Control measurements			FS reaches critical concentration values?
			Type	Nerve lesioning	Critical point	
Low cathodic (< 100 µA/cm^2^)	Leech	None	Hypoxia induction	None	N/A	N/A
			*H*_2_ *O*_2_ application	Confirmed	1 mm solution	No
	Locust	Confirmed within 30 min	Hypoxia induction	Confirmed	(21 ± 4) min	Yes (Pt only)
			*H*_2_ *O*_2_ application	Confirmed	70 µm solution	Yes (PEDOT only)
High cathodic (>100 µA/cm^2^)	Leech	Confirmed after 10–30 min	Alkaline solutions application	Confirmed	pH 11 solution	Yes
	Locust	Confirmed after 5–15 min	Alkaline solutions application	Confirmed	pH 9 solution	Yes
Anodic	Leech	Confirmed after 15–30 min	Acidic solutions application	Confirmed	pH 2 solution	No
			NaOCl application	Confirmed	14 mm ^a^ solution	Likely^b^
	Locust	Confirmed after 15 min	Acidic solutions application	Confirmed	pH 4 solution	No
			NaOCl solutions application	Confirmed	14 mm ^a^ solution	Likely^b^

^a^ These signify concentrations of the hypochlorite in the added bolus. Estimating real concentration actually reaching the nerve tissue is difficult due to the rapid reaction of hypochlorite with numerous substrates.

^b^ Electrochemical hypochlorite evolution on platinum in the voltage ranges we tested does produce 0.15 mm hypochlorite when measured in pure phosphate‐buffered saline, i.e., in a situation where degradation of the hypochlorite is not so rapid. Therefore, it is likely that these, or higher, concentrations are produced at the interface between the electrode surface and nerve.

### 3.1 Electrochemical Hypoxia via ORR as a Low‐Current Lesioning Mechanism

Our results show that low cathodic current densities (<100 µA/cm^2^) are sufficient to abolish neural conduction in the locust N5 nerve. In situ oxygen measurements and nitrogen‐sparging positive controls confirm that oxygen reduction reaction (ORR) drives local hypoxia severe enough to silence axonal transmission. The absence of measurable pH changes at these polarization levels further supports hypoxia, rather than alkalization or electric‐field effects, as the primary driver of conduction failure. The contrasting behavior of the leech nerve cord—fully resistant to the same cathodic currents and to externally applied hypoxia—provides a strong biological control. If lesioning were mediated by nonspecific electric‐field effects or other redox species, both models should behave similarly. Their divergence therefore validates ORR‐induced hypoxia as a distinct lesioning pathway.

### 3.2 2‐Electron Electrochemical ORR With Hydrogen Peroxide has Higher Current Efficiency

The comparison between platinum and PEDOT:PSS electrodes reveals that electrochemical hypoxia is not the only mechanism by which ORR can damage tissue. ORR occurs on PEDOT:PSS with substantially lower absolute current than on platinum, although functional nerve failure times were similar to those of platinum. This efficiency arises from the higher faradaic efficiency of ORR, when compared with Pt. The 2e^−^ ORR pathway consumes O_2_ twice as effectively per unit charge, and a large fraction of the current on Pt is wasted on HER. However, the most salient aspect of the 2e^−^ process on PEDOT:PSS is the formation of hydrogen peroxide, a cytotoxic species. Our positive controls show that peroxide levels plausibly produced by PEDOT:PSS electrodes (for recent quantification of PEDOT:PSS peroxide production in biological electrolytes, see the study from Jakešová et al. [32]) can damage insect nerves, supporting the view that peroxide‐induced stress may coexist with hypoxic stress. When we performed experiments of adding catalase or pyruvate during ORR on PEDOT:PSS, we found that the lesioning effect was eliminated and nerve function remained unchanged. Catalase and pyruvate both serve to neutralize hydrogen peroxide. Therefore, we conclude that hydrogen peroxide is the dominant mechanism of nerve failure caused by ORR. Therefore, PEDOT:PSS has a higher current efficiency than Platinum to cause functional lesioning, and this is probably to do with the effect of peroxide. Importantly, for both Pt or PEDOT:PSS, ORR‐mediated lesioning occurs at currents 10–100× lower than those needed to reach the water electrolysis regime. This identifies electrochemical hypoxia and hydrogen peroxide generation as previously underappreciated mechanisms of neural injury within the range of currents often considered “safe.”

### 3.3 Water Electrolysis and Alkalization as Higher‐Current Lesioning Modes

At more negative potentials, cathodic water electrolysis produces rapid and substantial alkalization. In both models, we observed pH elevations sufficient to disrupt conduction within minutes. Control experiments using alkaline buffers reproduced this lesioning threshold, confirming pH as the dominant mechanism. The leech results clarify an additional point: because the leech is resistant to hypoxia and peroxide stress, its vulnerability under high‐current cathodic stimulation directly implicates hydroxide ion accumulation, not oxygen depletion or reactive oxygen species, in the observed nerve failure. The convergence of locust and leech responses in this regime provides strong cross‐model validation for the chemical nature of alkalization‐based injury. The pH increase mechanism has been implicated in previous studies as a cause of tissue lesioning; its in situ measurement directly in a nerve lesioning model has not been reported before. Our work establishes the voltages and current densities that correspond to the cathodic alkalization regime.

### 3.4 Reactive Chlorine as a Major Contributor to Anodic Lesioning

Anodic DC produced nerve lesioning in both animals without appreciable local acidification, despite current densities sufficient to drive water oxidation (OER). This observation, together with our positive controls using hypochlorite, strongly implicates chloride oxidation—and the associated formation of reactive chlorine species (RCS)—as the key anodic lesioning pathway.

RCS such as hypochlorous acid are highly reactive, short‐lived oxidants that rapidly degrade lipids, proteins, and membranes. Their extreme reactivity explains both the rapid onset of lesioning and the difficulty of quantifying in situ concentrations. The fact that both species—locust and leech—showed similar susceptibility aligns with the expected general cytotoxicity of chlorine‐based oxidants. Importantly, these findings challenge the prevailing assumption that anodic DC damage arises primarily from acidification and suggest that chlorine chemistry, not pH, represents the true safety boundary during anodic electrochemical stimulation. We recently reported analysis of the coexistence of both OER and RCS forming reactions on platinum electrodes in the same potential window [20].

### 3.5 Implications for Tissue‐Interfaced Bioelectronics and Study Limitations

This work highlights that the conventional “water window”—defined by the onset of water splitting—is insufficient to predict tissue safety during DC application. Electrochemical hypoxia and RCS production occur at far lower potentials and currents than those associated with water electrolysis and associated pH changes, introducing new considerations for experimental design and allowable charge densities. These findings are important for interpreting DC stimulation experiments in vitro or in vivo, and safety in implantable neural interfaces.

Conversely, the Faraday Scalpel concept offers opportunities for precision, chemical‐based neurosurgical interventions. Unlike thermal ablation or high‐field electroporation, electrochemical lesioning operates at microwatt power levels, can be localized via electrode geometry, and can potentially target specific compartments (e.g., axons) without damaging surrounding vasculature. Unlike Platinum, PEDOT:PSS enables the mechanism of sustained hydrogen peroxide production via ORR, and a recent study from our group [32] found that PEDOT:PSS is completely stable under sustained cathodic polarization, and can reproducibly deliver hydrogen peroxide over many hours of operation. While our current study adds to evidence to the growing consensus that PEDOT:PSS electrodes can be stable, the remarkable capacity to produce cytotoxic levels of hydrogen peroxide raises potential safety concerns in neural interface electrodes intended for stimulation and not lesioning.

Our work also identifies limitations. Concerning the ORR effects of electrochemical hypoxia, or peroxide generation, their impact will heavily depend on tissue oxygenation and perfusion. The notion of electrochemical hypoxia was first described by Wong et al. in 2022 [31] in the context of in vitro experiments on neurons. In these experiments, axonal regions were subjected to electrochemical hypoxia, while the cell bodies remained in normoxic conditions. It was found that this induced apoptosis in cells. This indicates that exposing axonal segments to hypoxia may lead to overall cell death of the neuron, though this must be subjected to further study in vivo. Our results reported here do indeed fit into this axonal hypoxia injury model. Future mammalian studies will be essential to evaluate how vascular perfusion influences oxygen depletion dynamics and to determine whether electrochemical hypoxia can be leveraged for fine‐scale neuromodulation or targeted ablation in vivo. Conversely, the effects of ORR‐produced hydrogen peroxide will similarly need to be evaluated in specific mammalian tissues, where perfusion and presence of antioxidant enzymes will modulate the effects that hydrogen peroxide will have. In these invertebrate models, we did not perform a histological assessment of cellular injury, making it difficult to definitively distinguish between transient nerve block and irreversible cell death. However, the absence of recovery over 48–72 h and the consistency with recent in vitro studies of electrochemical hypoxia strongly support the interpretation of true lesioning rather than reversible block. Moreover, the translation of these findings to mammalian systems must account for differences in tissue perfusion, antioxidant capacity, and anatomical scale. We believe that investing in detailed histological investigation should be done during experiments on mammalian models. Another significant limitation is understanding the effects of reactive chlorine species (RCS) produced by anodic chloride oxidation. Unlike with oxygen or hydrogen peroxide, in situ quantification of hypochlorite is not straightforward [20, 35]. Moreover, RCS reacts extremely rapidly. Recent studies have established that electrochemically‐generated RCS can be highly cytotoxic and used as an anticancer therapy [36]. Future work needs to be directed to understand the mechanism of action of RCSs in nervous tissue. Development of selective in situ electrical probes in vivo would be useful for resolving RCS mechanisms [35].

Despite these limitations, our study shows mechanistic clarity in terms of how Faradaic lesioning can work, identifying discrete electrochemical mechanisms. While pH effects in lesioning are not a new idea, we believe electrochemical hypoxia and/or ROS and reactive chlorine species are novel mechanisms in this field, which can serve as both a caveat and an inspiration to a diverse range of bioelectronics applications.

## 4 Experimental Methods

### 4.1 Experimental Setup and Animal Handling

Experiments on leeches and locusts, as invertebrates, do not fall under legislation in the Czech Republic as animal experiments; no special ethical permission is required (Act No. 246/1992 Sb.). For testing the lesioning effect of the Faraday Scalpel concept on nerve tissue, locust and leech models have been established. Both of these models involve stimulation of a nerve structure and using evoked motor activity as a biomarker of nerve function and transmission of the evoked signal. Locusts: Adult specimens of *Locusta migratoria* were obtained at a local pet shop and housed in terraria with a heating lamp and ad libitum grass and oats. Leech: Adult specimens of medical leech (*Hirudo verbana*, large size) were obtained from a leech breeding farm (Etnomagic, Ústí nad Labem, Czech Republic). In line with the breeder's quality assurance guidelines, the leeches were housed in glass containers, filled to approximately two‐thirds volume with stale tap water sourced from Brno, Czech Republic. To maintain optimal water quality, two‐thirds of its volume was refreshed on a weekly basis. The containers holding the leeches were stored in a dark environment with room temperatures maintained between 20°C and 23°C.

### 4.2 Electrode Microfabrication

#### 4.2.1 Leech Electrodes

Flexible and peelable stimulation electrodes were fabricated on 4‐inch borofloat carrier wafers. We started with the surface treatment of the wafer using O_2_ inductively coupled plasma (ICP). This was followed by spin‐coating of the bottom substrate layer of polyimide (PI 2610, HDMicrosystems) at 2000 rpm to achieve a final thickness of 3 µm. The coated wafers were pre‐cured at 110°C for 4 min at ambient atmosphere and subsequently cured again in a tube furnace at 300°C in N_2_ atmosphere for 30 min with a ramp of 5°C∙min^−1^ while the pressure was ≈ 66 661 Pa. In the next step, we deposited 50 nm of low‐temperature AlN as a part of the bottom encapsulation using a dual Kaufman ion‐beam source setup, which was followed by deposition of the electrode material stack of 5 nm Ti/150 nm Pt/5 nm Ti without breaking the vacuum. Then, the first lithography to obtain the shape of leads, pads, and electrodes was performed with positive photoresist, followed by dry etching. The top Ti was etched with SF_6_/Ar capacitively‐coupled plasma (CCP) reactive ion etching (RIE). Then, Ar ion‐milling was used to sputter Pt, and with etch‐stop detection at the bottom of Ti, which was then etched in the CCP RIE instrument. That allowed stopping the etch at the bottom AlN layer without damaging it. These steps were followed by photoresist stripping in a DMSO‐based solution MLO‐07 (AZ) at 80°C. Then, the wafers were rinsed with isopropyl alcohol (IPA) and DI water prior to O_2_ treatment before deposition of the top encapsulation layer. O_2_ plasma treatment was performed in the same conditions as described above. Then, wafers were ready for the deposition of the top encapsulation layer, which started with the deposition of AlN with the same parameters as we used for the bottom layer to eliminate the stress gradient. After the AlN deposition, O_2_ treatment was performed to oxidize the surface of AlN, which is critical for adhesion of the top PI layer mediated by adhesion promoter (VM‐651, HDMicrosystems). Deposition and curing of the PI top encapsulation layer were done with the same parameters as used for the bottom PI layer. Strict maintenance of the same parameters is important to avoid undesired stress gradients, which can cause unwanted bending of devices. The next lithography step with positive resist was focused on the opening of pads and electrodes. After resist development, the PI layer was etched in the RIE using O_2_ and SF_6_ ions produced by ICP and accelerated by a high‐frequency plasma generator, and the AlN layer acted as an etch‐stop. The AlN and the top Ti layer were etched using BCl_3_ and Cl_2_ plasma with optical spectrometer endpoint detection to stop at the Pt surface and minimize the sputtering of the material. Then, the resist was stripped and the wafer cleaned prior to the final lithography. The aim of the last lithography step was to create trenches around devices to make them peelable. For that purpose, positive PR was used to define the trenches, and the PI and AlN layers were etched with the RIE as described above. In the last step, the PR was stripped, making the electrodes ready for the last peel‐off process, which was done by adding water droplets to delaminate the electrode prior to use. Monopolar FS electrodes had an active area of 2 × 5 mm, while the bipolar stimulation electrodes were each 2 × 3 mm.

#### 4.2.2 Locust Electrodes

Flexible microelectrodes were fabricated on 4‐inch Borofloat wafers coated with a 2 µm layer of parylene‐C deposited in an SCS Labcoater 2. Each device consisted of four electrodes with an active area of 1000 × 100 µm. The surface was activated in oxygen plasma (Diener NANO Plasma Cleaner) before depositing a 20/30/20 nm Ti/Au/Ti metal stack by e‐beam evaporation (Bestec). Metallization was patterned by the first photolithography step (AZ1514H), followed by descum in oxygen plasma. The Ti and Au layers were then patterned by wet etching (HF/H_2_O_2_/H_2_O for Ti and KI/I_2_ for Au), and the remaining resist was stripped in acetone. The surface was activated again in oxygen plasma to enhance adhesion of the second, encapsulating 2 µm parylene‐C layer, with A‐174 silane adhesion promoter introduced directly into the deposition chamber. A second photolithography step (AZ1518) defined the electrode and pad openings, which were etched into the top parylene‐C film by oxygen plasma reactive ion etching (PlasmaPro 80 RIE, Oxford Instruments). The exposed Ti layer was removed by wet etching, and after oxygen‐plasma activation, a 10 nm platinum layer was deposited by magnetron sputtering (Bestec) and patterned by lift‐off in acetone. Device outlines were defined in a third lithography step (AZ12XT) and etched through both parylene‐C layers using oxygen plasma RIE. After the final resist stripping, the devices were finished; removal from the wafer was facilitated by introducing a small drop of water along the outline and gently lifting the structures using fine forceps or a soft brush.

Fabrication of PEDOT:PSS‐based electrodes followed the same process sequence up to the second lithography step. At that point, only the contact pads were opened in the parylene layer, and the underlying Ti was selectively etched to expose the Au layer, ensuring low‐resistance electrical contact with the connector. The device outlines were defined and etched as described above. Following resist removal, an additional sacrificial 2 µm parylene‐C layer was deposited, preceded by spin‐coating of an anti‐adhesion film of 2% Micro‐90 solution (1000 rpm, 1 min). A fourth lithography step (AZ12XT) opened the electrode regions, and the upper parylene layers were etched just enough to expose and activate the Ti surface, which serves as an adhesion layer for PEDOT. Excessive oxidation of Ti during RIE was avoided, as it increases PEDOT–Ti contact resistance. The conductive polymer layer was deposited by spin‐coating a freshly prepared solution of PEDOT:PSS (PH1000; Heraeus GmbH) containing 5 wt.% dimethyl sulfoxide (DMSO), 0.1 wt.% 4‐dodecylbenzenesulfonic acid (DBSA), and 2 wt.% (3‐glycidyloxypropyl)trimethoxysilane (GOPS). The PEDOT:PSS/DMSO/DBSA mixture was sonicated for 15 min, GOPS was added immediately before use, and sonicated for an additional 2 min. The final solution was filtered through a 0.45 µm PVDF membrane and spin‐coated at 1000 rpm, followed by a 30 s bake at 80°C. After drying, the sacrificial parylene‐C layer was peeled off to expose the PEDOT:PSS electrodes, which were finally annealed at 120°C for 30 min.

### 4.3 Locust Preparation

Prior to preparation, the adult locust was anesthetized by refrigeration for approximately 5 min. Subsequently, the locust was positioned dorsal side up on a plasticine support and immobilized to allow only hind leg movement. A rectangular incision (approximately 5 × 3 mm) was made in the metathoracic region to expose the metathoracic ganglion together with bilateral N5 nerves, which innervate the hind legs. Furthermore, the surrounding dense tracheal network was carefully removed, leaving only the main tracheal trunks intact.

### 4.4 Treatment of the N5 Locust Nerve by the Faraday Scalpel

A thin, flexible electrode, fabricated on parylene‐c and incorporating 4 active electrode sites (platinum or PEDOT:PSS), was used. The electrode was inserted above the tracheal trunks and positioned beneath the N5 nerve, so that the nerve rested directly on the electrode's active sites. A custom‐designed printed circuit board adapter was used to establish electrical connections between the electrode and the recording and stimulation apparatus. The first two electrode sites were designed for nerve stimulation, driven by a waveform generator (EDU33212A, Keysight) coupled to a biphasic current stimulator (DS4, Digitimer Ltd.). Stimulation consisted of a cathodic‐leading biphasic square waveform with a pulse width of 250 µs, delivered periodically at 1‐min intervals. Stimulation current was set at the start of each experiment to the threshold for extension of the leg via stimulation of the *extensor tibialis* muscle. A direct current was supplied to either of the two remaining electrode sites via a potentiostat (PalmSens4, PalmSens, Houten, Netherlands). A gold wire, implanted in the abdominal cavity of the locust, remote from the treated site, served as a counter electrode, while an Ag/AgCl reference electrode was positioned within the metathorax near the active electrode. During DC treatment, hind leg movements were monitored following each stimulation, and the time point at which the response ceased was recorded. Current densities are reported with respect to the geometric electrode area, all of which is immersed in locust lymph and thus electrochemically active. Only a fraction of this total electrode area is in contact with the N5 nerve, however. For experiments with PEDOT:PSS electrodes, to discriminate the role of hydrogen peroxide, two peroxide‐neutralizing protocols were tried: (1) Adding 5 µL of 200 µm pyruvate solution, or (2) Adding 5 µL of 8500 U/mL catalase solution. These solutions were injected directly over the N5 nerve/electrode region.

### 4.5 Treatment of the N5 Nerve in Positive Controls: Hypoxia, Hydrogen Peroxide, Acidic, Alkaline, or HOCl Solutions

To investigate the effects of different damaging conditions on N5 nerve integrity, several control treatments were applied. For hypoxia experiments, a fine rubber tube delivering moisturized nitrogen gas was directed into the metathoracic cavity, after which the opening was sealed with a plastic film to maintain a closed environment. Nitrogen was continuously supplied, and changes in hind leg responses to mechanical stimulation of the abdomen were monitored throughout the treatment. An air‐supplied experiment was also performed as a control. For liquid solution experiments, a segment of the N5 nerve was isolated from the surrounding tissues using 3 plastic foil strips: two placed above adjacent structures and one below the nerve. Test solutions (hydrogen peroxide, acidic, alkaline, or HOCl Chlorine bleach) were applied to the exposed nerve segment. The duration of exposure and applied concentrations were systematically recorded, along with any changes in hind leg movement following periodic mechanical stimulation of the abdomen during the treatment. The solutions were prepared from insect medium: (Insect medium consisted of 140 mm NaCl, 8 mm KCl, 2.3 mm CaCl_2_, and 10 mm HEPES, 90 mm sucrose, 5 mm glucose. The pH of the solution was adjusted to 7.2.)

### 4.6 Semi‐Intact Leech Preparation

To prepare the semi‐intact leech preparation, the leech was first anesthetized by immersion in crushed ice for 15 min. Then, the head ganglion and sucker were excised to minimize variability and suppress spontaneous activity. The leech was extended and secured ventral side up in a 120 mm stainless steel Petri dish, which had a PDMS (polydimethylsiloxane, Sylgard 184 (Dow Corning) substrate covered with a 3% agarose layer (Roth) dissolved in leech medium (Leech medium consisted of 115 mm NaCl, 4 mm KCl, 1.8 mm CaCl_2_, and 10 mm HEPES + Ca^2+^ + glucose. The pH of the solution was adjusted to 7.4) to enhance biocompatibility, wettability, and an effective electrolytic contact to the leech. Throughout the entire experimental duration, the leech was maintained in a moist environment. The caudal segment of the leech was sutured using surgical material employing a simple transverse loop and then attached to the arm of the force sensor. The anterior segment of the leech was elevated using a polypropylene pad, and a bipolar gold stimulation electrode was positioned on its skin surface. This setup ensured that the electrodes remained insulated from surrounding moisture so that the stimulation current path would be isolated to the anterior section of the animal.

To expose the nerve cord, a longitudinal incision, spanning the length of 5 ganglia, was made through both the skin and muscle layers using a scalpel blade No 11. Surrounding soft tissues were delicately dissected using fine scissors and forceps (Fine Science Tools, Foster City, California, USA), ensuring the preservation of bilateral nerves and the underlying gut. The black sheath over one ganglion was excised, and the bilateral nerve projections across three adjacent ganglia were severed. Subsequently, the 3D printed application chamber was gently slid beneath the cord and stabilized using fine pins, and the Faraday Scalpel electrode was then applied.

### 4.7 Treatment of the Nerve Cord by the Faraday Scalpel Electrode

The Faraday Scalpel was crafted from flexible polyimide, with an active layer of platinum as previously detailed. This electrode was securely mounted in a custom 3D‐printed stimulation chamber, which was placed beneath the cord right after finalization of the leech preparation procedure. The electrode installation involved threading it through a plastic guide loop in the chamber and carefully wrapping it around the nerve cord, and then guiding it back through the same plastic loop. Close tightening of the electrode around the cord was then accomplished by tightening a locking bolt that is built into the chamber. For establishing a connection between the scalpel electrode and the DC power supply, we used a specially designed printed circuit board adapter equipped with Zero Insertion Force (ZIF) clips. The DC current was supplied by a potentiostat (PalmSens4, PalmSens, Houten, Netherlands), operated in galvanostatic mode. A gold wire was placed at the edge of the petri dish, in contact with the leech medium, and this served as a counter electrode. Meanwhile, a reference electrode was placed in the leech medium near the site of the Faraday scalpel electrode. All experiments were done in this three‐electrode arrangement controlled by the potentiostat. Current densities are reported with respect to the geometric electrode area, all of which is immersed in leech solution and thus electrochemically active. Due to the nerve cord/electrode chamber design, all of this electrode area is in contact with the nerve itself.

### 4.8 Treatment of the Nerve Cord by Positive Controls: Hypoxia, Hydrogen Peroxide, Acidic or Alkaline Leech Medium, or by NaOCl Solutions

To assess the impact of electrochemical changes resulting from Faraday Scalpel activity on the functional integrity of the nerve cord, specific cord treatment methods were employed. Following the completion of the leech preparation, a 3D‐printed chamber was positioned beneath the cord. This chamber featured a midline slit for the cord, with raised edges to retain the liquid inside, thus preventing leakage and safeguarding the exposed cord from drying.

For inducing hypoxia, the upper part of the chamber, outfitted with inlet and outlet vents for moisturized nitrogen or argon gas circulation, was utilized. To explore the effects of various possible electrochemical products, a range of solutions was introduced into the chamber. These included hydrogen peroxide freshly mixed with leech medium at varying concentrations (10 nm to 1 mm), leech medium either acidified with HCl or made alkaline using NaOH, or chlorine bleach (in the form of dissolved NaOCl), which was freshly diluted in leech medium immediately before application due to the rapid reactivity of chlorine.

### 4.9 Leech Stimulation and Recording

The stimulation was conducted using an alternating current delivered via bipolar electrodes. A specialized, automated in‐house system—comprising a waveform generator, current isolator, and Arduino microcontroller—was utilized to ensure consistency in the stimulation process throughout the experiment. The stimulation pulse was a cathodic‐leading biphasic square wave, 5 ms per phase, and was generated by an EDU33212A waveform generator (Keysight). This voltage waveform was relayed to the DS4 current stimulator (Digitimer Ltd.), which delivered current to the flexible bipolar stimulation electrode on the leech. The connection between the electrode and the current isolator was facilitated by a custom‐made printed circuit board adapter fitted with ZIF clips. To generate a stimulus train, trigger impulses from an Arduino Nano directed the waveform generator, determining both the number of pulses in a single train and the intervals between them. Concurrently, a parallel signal was dispatched to the digital port of the Intan USB interface board (Intan Technologies, Los Angeles, California, USA), acting as a trigger for subsequent automated data analysis.

The recording component of the setup incorporated a standard load cell (range 0–100 g, Eclipsera s.r.o., Havlíčkův Brod, Czech Republic) connected to another Arduino Nano. The load cell signal was digitized by a 24‐bit, 2‐channel analog‐to‐digital converter (HX711, Eclipsera s.r.o.), processed by the Arduino Nano, and displayed in real‐time using the serial plotter of the Arduino IDE software. Simultaneously, this signal was converted back to analog by a digital‐to‐analog converter (PCF8591, Eclipsera s.r.o.) and transmitted to the same Intan USB board that received the stimulation trigger signal. The data were collected by an Intan USB board and stored in a common RHD file until further processing.

## Funding

HORIZON EUROPE European Research Council 949191; Ministerstvo Školství, Mládeže a Tělovýchovy LX22NPO5107; Grantová Agentura České Republiky No 23‐07432S.

## Conflicts of Interest

The authors declare no conflicts of interest.

## Supporting information




**Supporting File**: advs74857‐sup‐0001‐SuppMat.docx.

**Supporting File**: advs74857‐sup‐0002‐Data.zip.

## Data Availability

The data that support the findings of this study are available in the supplementary material of this article.
