# Supplementary material for: The Faraday Scalpel: Electrochemical Nerve Lesioning Mechanisms Studied in Invertebrate Models
Source: Adv Sci (Weinh). 2026 Mar 17;13(30):e23797. doi: 10.1002/advs.202523797 (PMC13248754; doi:10.1002/advs.202523797)
Supplement: Supplementary file 1 — Supporting File: advs74857‐sup‐0001‐SuppMat.docx. [file ADVS-13-e23797-s001.docx]

Supporting Information

**The Faraday Scalpel: Electrochemical nerve lesioning mechanisms studied in invertebrate models**

Petra Ondráčková, Jan Švec, Marie Jakešová, Jiří Ehlich, Imrich Gablech, Eric Daniel Głowacki*


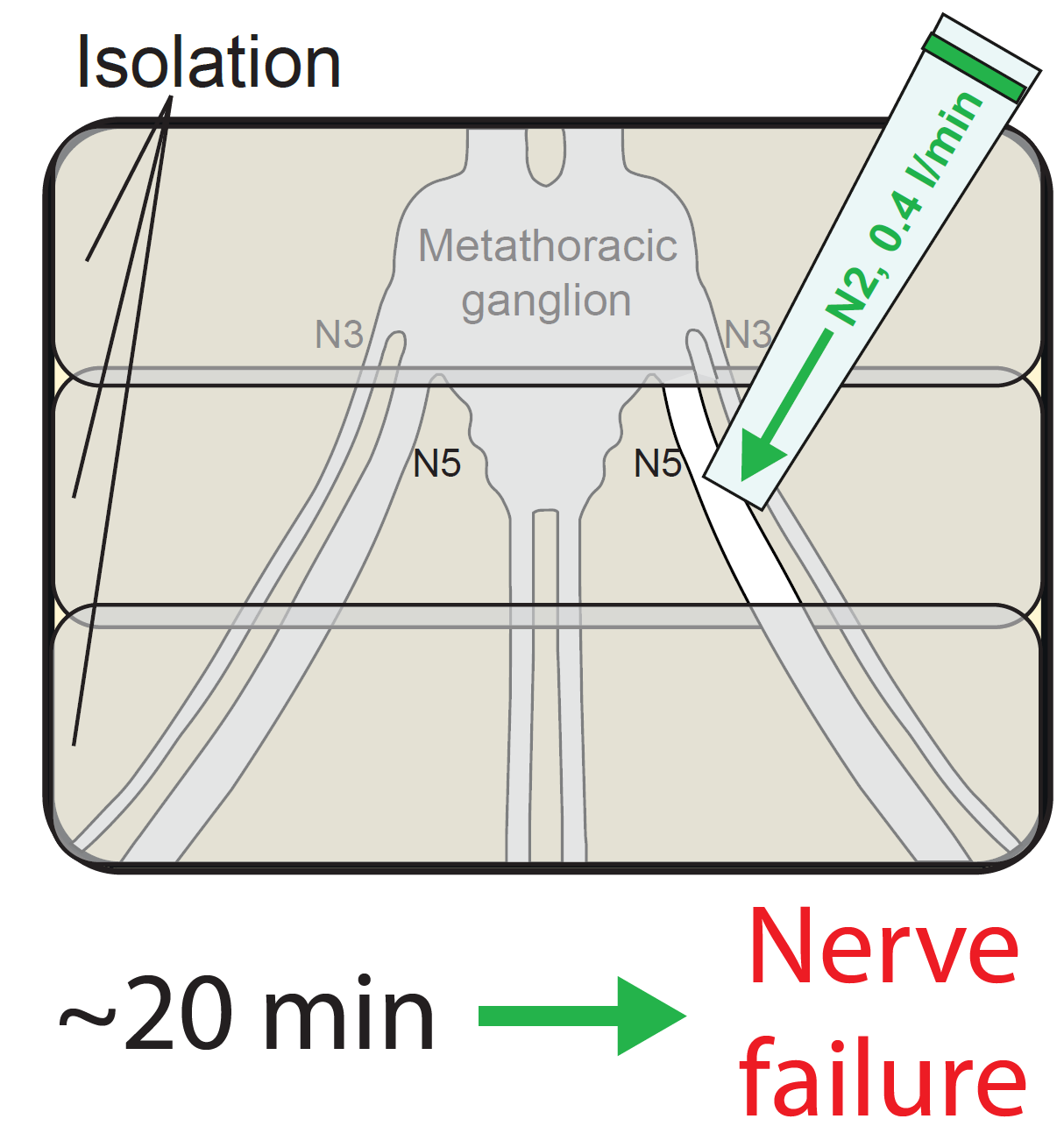

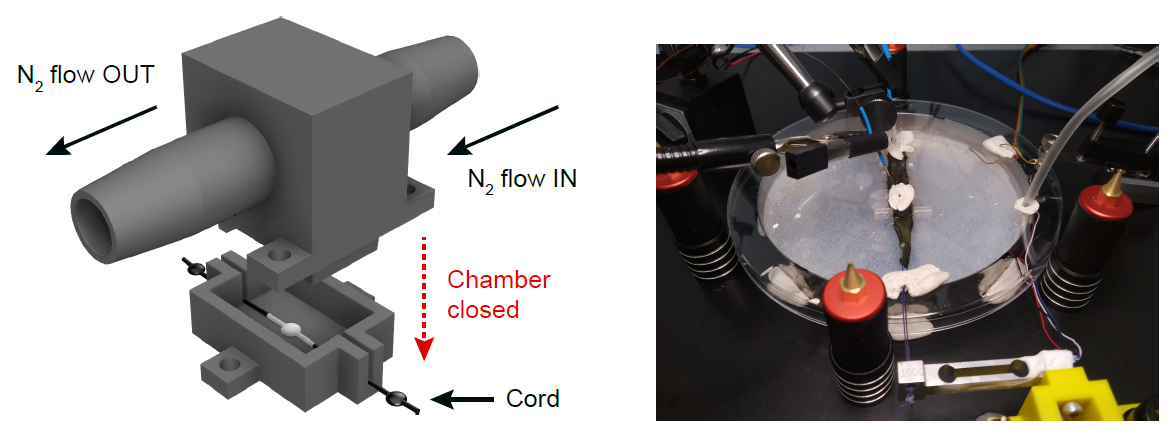


**Figure S1.** Hypoxia positive-control experiments. The schematic on the left shows the experimental setup for the locust: A test portion of the locust N5 nerve was exposed to a flow of moist N_2_ gas, while strips of parylene-c foil were used to isolate other parts of the exposed metathorax. As expected from what is known about insect sensitivity to hypoxia, nerve failure occurred within 20 min of hypoxia induction. The center and right-hand image show how hypoxia experiments were carried out in the leech. A 3D-printed chamber was used to isolate a test portion of the nerve cord. Even after 60 min of applying N_2_ or Ar gas, no nerve failure was observed, consistent with expectations on what is known about leech resistance to hypoxia. We even tried an experiment with the whole leech under an argon blanket (image on the right), and we did not observe any detriment to nervous function in this animal.


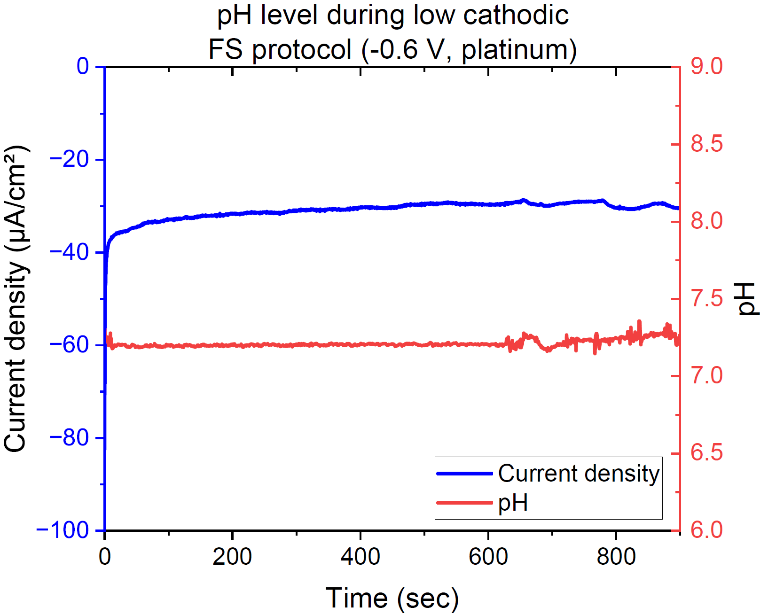


**Figure S2.** Measurement of local pH during application of low cathodic FS protocol in a locust. No alkalization is measured.


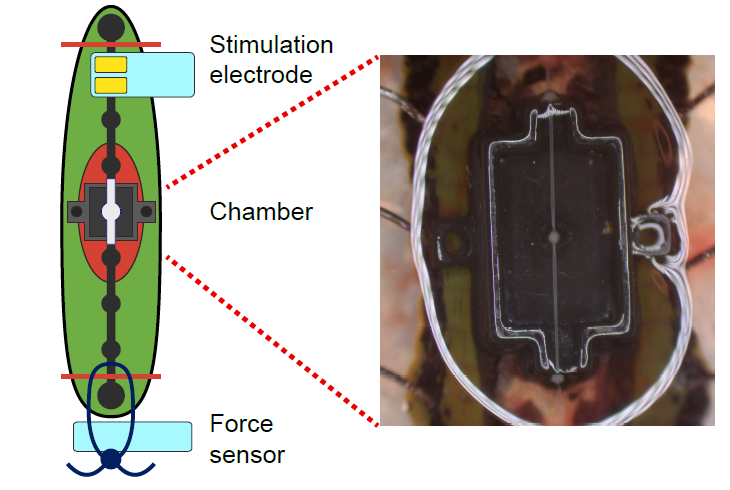

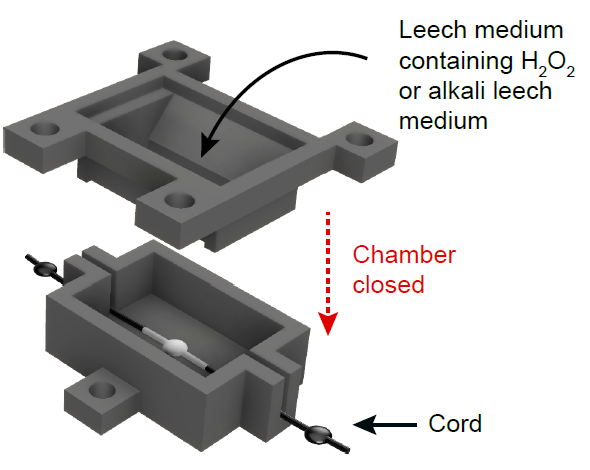

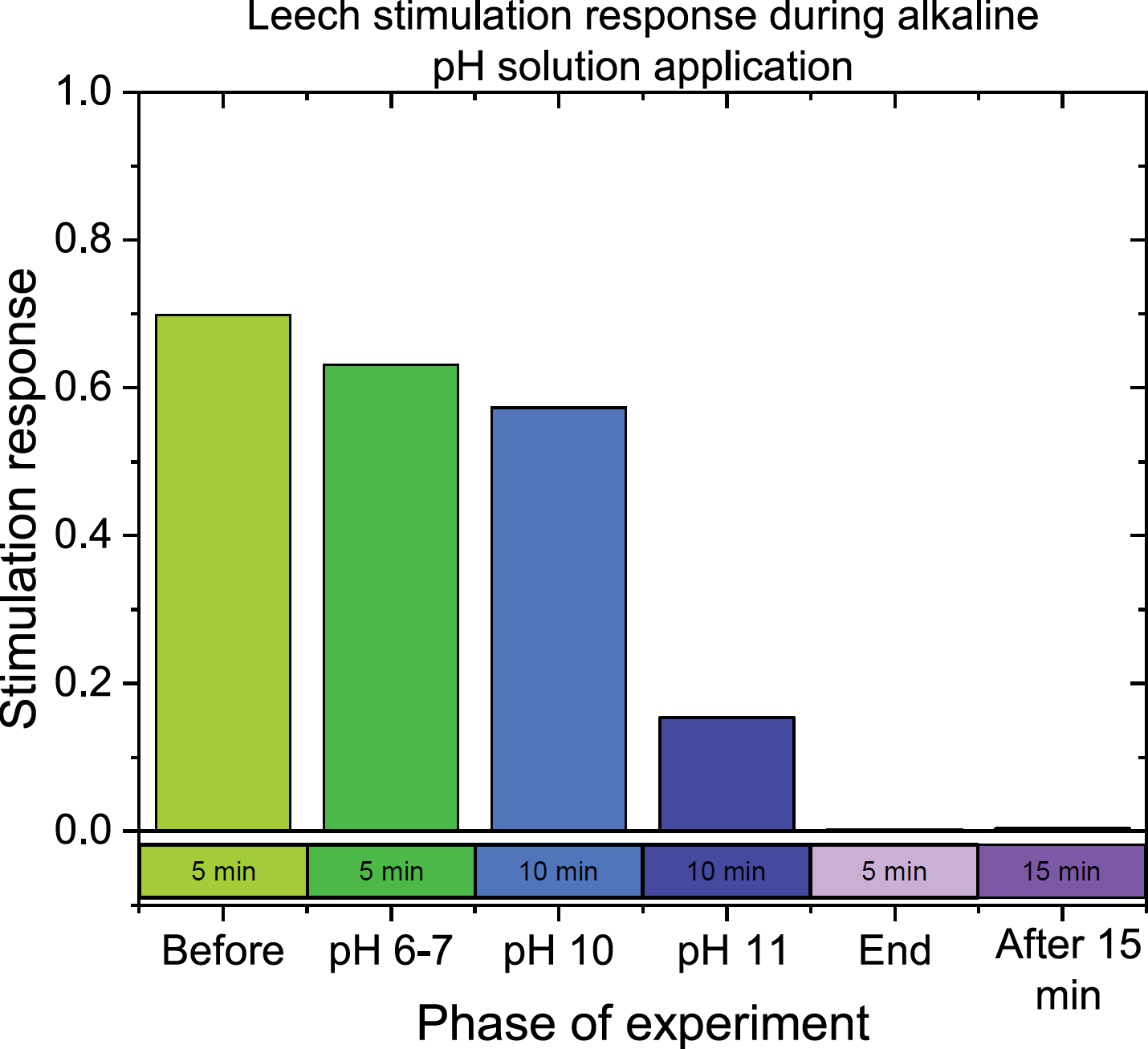


**Figure S3.** Control experiments on exposing the leech nerve cord to alkali media. The test portion of the nerve cord was isolated with a 3D-printed pool to allow controlled exposure of the test section to a given solution. The graph shows the decreasing evoked response as increasingly-alkali media are added to the pool.


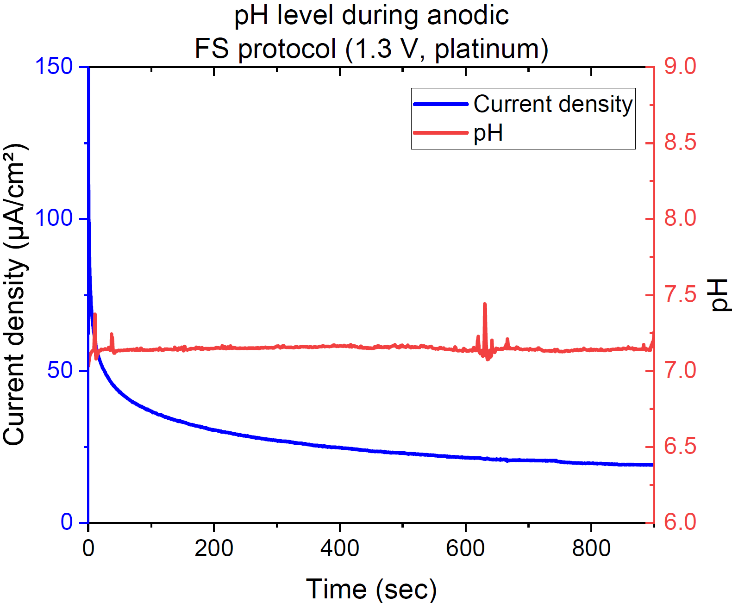


**Figure S4.** Measurement of local pH during application of anodic FS protocol in a locust. No acidification is measured.


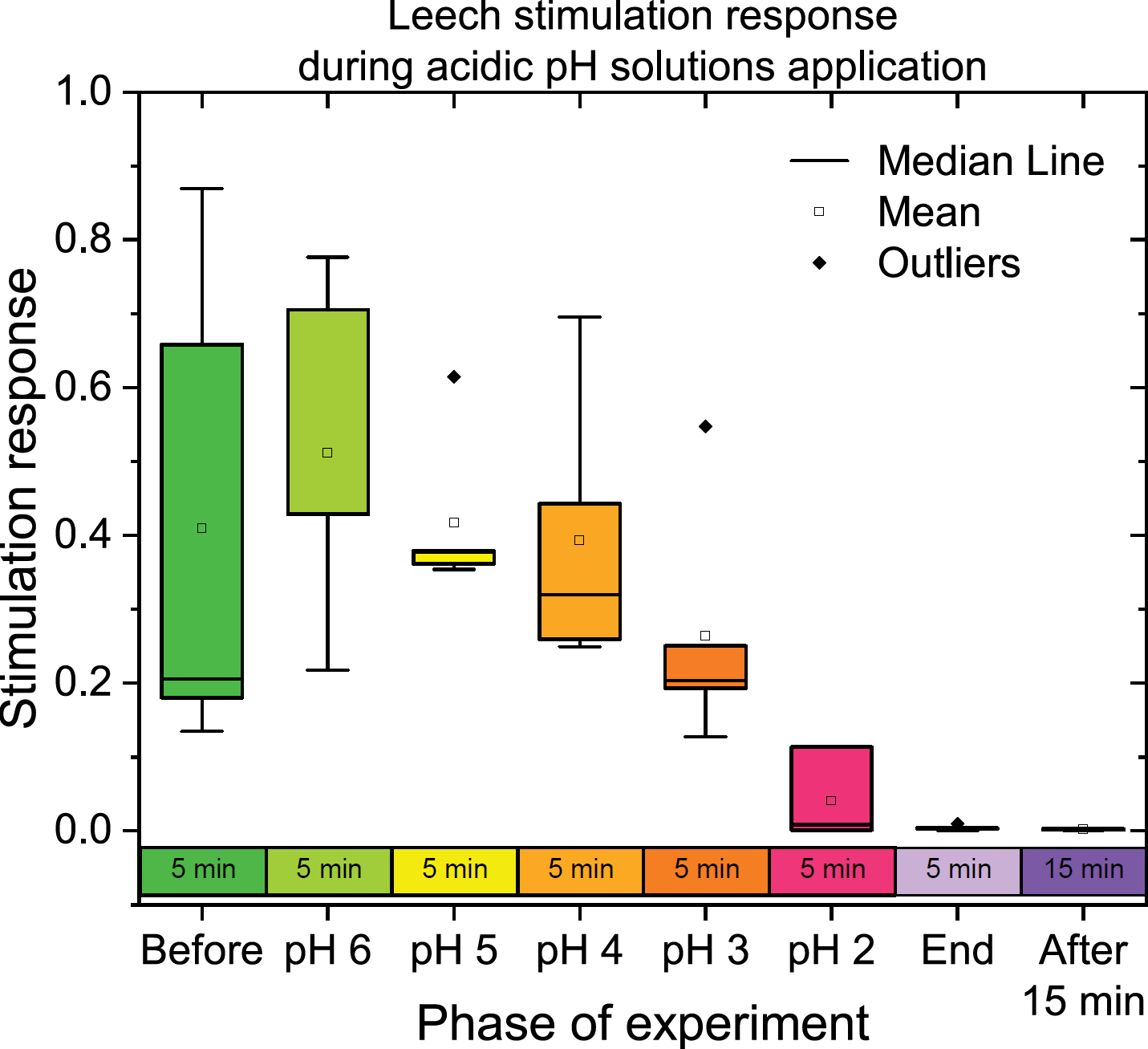


**Figure S5.** Positive control experiment of the leech nerve cord response to acidic pH. The experimental setup is the same as shown in Figure S3.


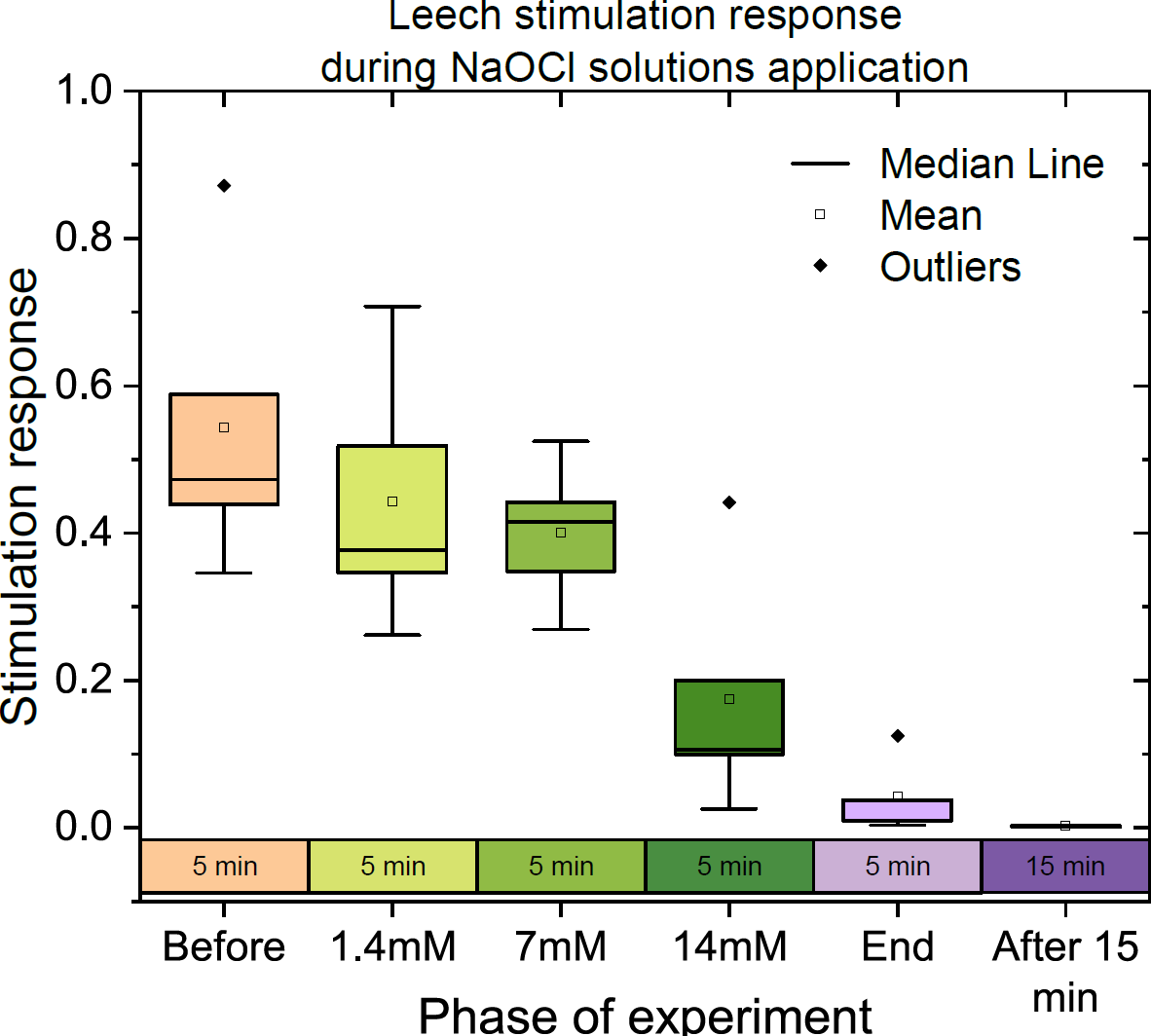


**Figure S6.** Positive control experiment of the leech nerve cord response to chlorine bleach solution. The experimental setup is the same as shown in Figure S2. The concentrations of NaOCl are based on conducting an assay on the solution before the application experiment, however the degradation of NaOCl upon contact with biological media is so rapid, that the actual concentration reaching the nerve is impossible to accurately estimate.
